# Synthetic slip plane, a hybrid kind of calcite twin data in dynamic analysis

**DOI:** 10.1016/j.mex.2019.02.004

**Published:** 2019-02-12

**Authors:** Yehua Shan, Jian Zheng, Xinquan Liang

**Affiliations:** a Laboratory of Marginal Sea Geology, Guangzhou Institute of Geochemistry, Chinese Academy of Sciences, Guangzhou 510640, China; b University of Chinese Academy of Science, Beijing 100049, China; c State Key Laboratory of Isotope Geochemistry, Guangzhou Institute of Geochemistry, Chinese Academy of Sciences, Guangzhou 510640, China

**Keywords:** Synthetic slip plane, Inversion, Reduced stress, Calcite, Twinned/untwinned e-plane

## Abstract

This paper defines a synthetic slip plane as the linear combination of a pair of twinned and untwinned e-planes in a single calcite crystal. This auxiliary slip plane is dependent upon neither the twinned e-plane nor the untwinned e-plane. It can be used together with the twinned e-plane either to further constrain the extents of the compression and tension axes in graphic dynamic analysis or to better estimate the reduced stress in numerical dynamic analysis.

Highlights

• The linear combination of twinned and untwinned e-planes in a calcite crystal defines the synthetic slip plane.
• The synthetic slip plane can be incorporated into graphic and numerical dynamic analysis.

## Graphical abstract

The synthetic slip plane is defined as the linear combination of a pair of twinned and untwinned e-planes in a single calcite crystal. This new kind of calcite e-twin data can be used together with the twinned e-plane to better determine stress in dynamic analysis.

**Figure:**
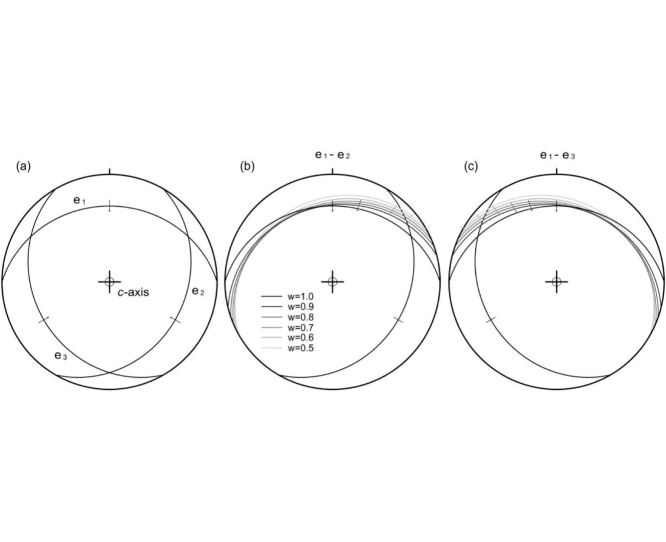

Specifications table

**Table:** 

**Subject Area**	• *Earth and Planetary Sciences*
**More specific subject area:**	*Structural geology*
**Method name:**	*Synthetic slip plane*
**Name and reference of original method**	*No*
**Resource availability**	*No*

## Method details

Calcite e-twin data have been inverted for stress in rocks since the pioneering work of Turner [1]. This becomes an important tool for structural geologists to quantify stress in the upper crust during geological history [2,3]. Various methods of this dynamic analysis have been developed. They are based upon two kinds of e-twin data: twinned e-planes [1,4,5], and both twinned e-planes and untwinned e-planes [[6], [7], [8], [9], [10], [11], [12]]. In this paper we introduced a new kind of e-twin data, synthetic slip plane, as the linear combination of a pair of twinned and untwinned e-planes in a single calcite crystal. It can be used together with the twinned e-plane to better determine stress in dynamic analysis.

## Synthetic slip plane

**Fig. 1:**
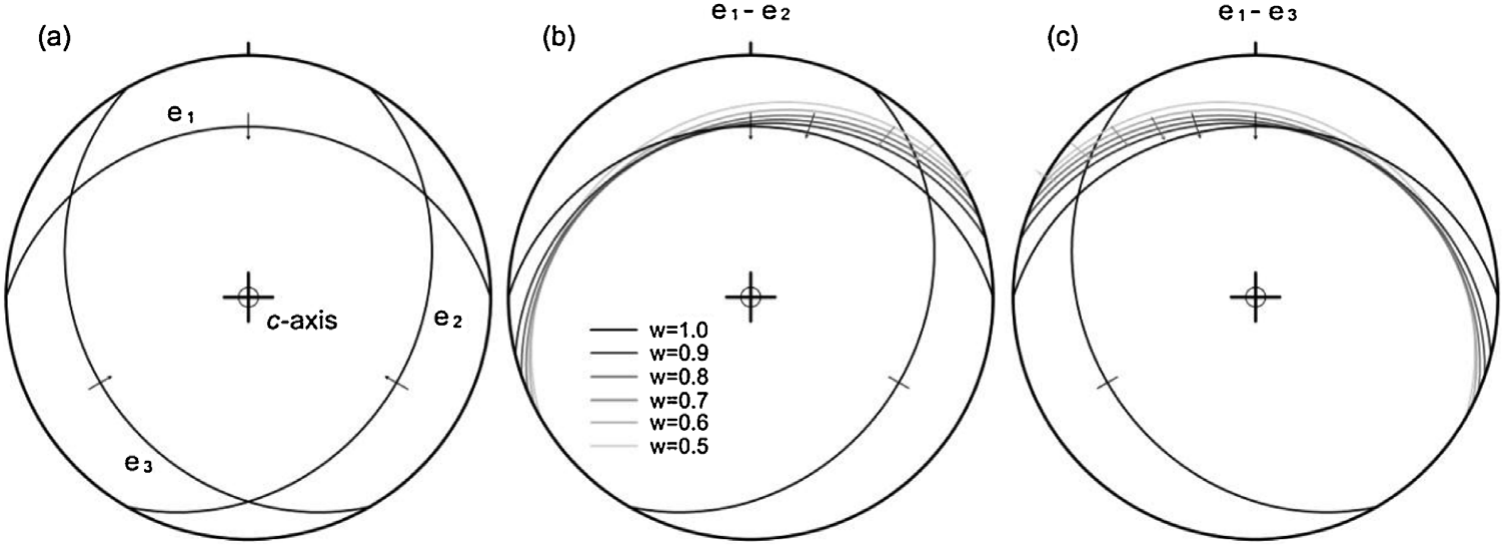
(a) The e-planes and gliding directions in a single calcite with an upright *c*-axis. The synthetic slip planes from (b) a pair of twinned (e_1_) and untwinned (e_2_) e-planes and (c) a pair of twinned (e_1_) and untwinned (e_3_) e-planes, respectively, in the crystal. Great circles represent e-planes or synthetic slip planes. Short lines mark the gliding directions or the slip lines, and arrows indicate the gliding or slip senses, for instance, reverse in this figure. Lower-hemisphere, equal-area projection.

Calcite mechanical twinning occurs along the *e* slip system (Fig. 1a), when the resolved shear stress on the system, τ, exceeds the critical resolved shear stress, $\tau_{c}$. Let us consider a pair of twinned (*e*_1_) and untwinned (*e*_2_) e-planes in a calcite crystal with an upright *c*-axis (Fig. 1b),

(1a) $$n^{\left(1\right)}\sigma s^{\left(1\right)}\geq \tau_{c}$$

and

(1b) $$n^{\left(2\right)}\sigma s^{\left(2\right)}<\tau_{c},$$

where σ is the unknown stress, $n^{\left(i\right)}$ (*i* = 1,2) is the normal vector to the *i*-th e-plane, and $s^{\left(i\right)}$ is the vector of the gliding line on the plane. $n^{\left(i\right)}=\left[\begin{array}{ccc} n_{1}^{\left(i\right)} & n_{2}^{\left(i\right)} & n_{3}^{\left(i\right)} \end{array}\right]$, and $s^{\left(i\right)}=\left[\begin{array}{ccc} s_{1}^{\left(i\right)} & s_{2}^{\left(i\right)} & s_{3}^{\left(i\right)} \end{array}\right]$, where $n_{3}^{\left(i\right)}\geq 0$ and, due to slipping or potentially slipping along the twinned or untwinned e-plane towards the *c*-axis, $s_{3}^{\left(i\right)}\leq 0$. Both the vectors have a unit length.

Weighting these inequalities and subtracting the second inequality from the first inequality,

(2) $$wn^{\left(1\right)}\sigma s^{\left(1\right)}-\left(1-w\right)n^{\left(2\right)}\sigma s^{\left(2\right)}\geq 0$$

where *w* is weight, and $w\in \left[\begin{array}{cc} 0.5 & 1 \end{array}\right]$.

Recasting the above inequality in Fry’s [13] full stress space,

(3) $$\left\{w{\left[\begin{array}{l} n_{1}^{\left(1\right)}s_{1}^{\left(1\right)}\\ n_{1}^{\left(1\right)}s_{2}^{\left(1\right)}+n_{2}^{\left(1\right)}s_{1}^{\left(1\right)}\\ n_{1}^{\left(1\right)}s_{3}^{\left(1\right)}+n_{3}^{\left(1\right)}s_{1}^{\left(1\right)}\\ n_{2}^{\left(1\right)}s_{2}^{\left(1\right)}\\ n_{2}^{\left(1\right)}s_{3}^{\left(1\right)}+n_{3}^{\left(1\right)}s_{2}^{\left(1\right)}\\ n_{3}^{\left(1\right)}s_{3}^{\left(1\right)} \end{array}\right]}^{T}-\left(1-w\right){\left[\begin{array}{l} n_{1}^{\left(2\right)}s_{1}^{\left(2\right)}\\ n_{1}^{\left(2\right)}s_{2}^{\left(2\right)}+n_{2}^{\left(2\right)}s_{1}^{\left(2\right)}\\ n_{1}^{\left(2\right)}s_{3}^{\left(2\right)}+n_{3}^{\left(2\right)}s_{1}^{\left(2\right)}\\ n_{2}^{\left(2\right)}s_{2}^{\left(2\right)}\\ n_{2}^{\left(2\right)}s_{3}^{\left(2\right)}+n_{3}^{\left(2\right)}s_{2}^{\left(2\right)}\\ n_{3}^{\left(2\right)}s_{3}^{\left(2\right)} \end{array}\right]}^{T}\right\}\left[\begin{array}{l} \sigma_{11}\\ \sigma_{12}\\ \sigma_{13}\\ \sigma_{22}\\ \sigma_{23}\\ \sigma_{33} \end{array}\right]=v^{\left(0\right)}\cdot \bar{\sigma }>0$$

where $v^{\left(0\right)}$ is the synthetic datum vector in the six-dimensional stress space, $\bar{\sigma }$ is the stress vector, and T is matrix transposition. $v^{\left(0\right)}=\left[\begin{array}{cccccc} v_{1}^{\left(0\right)} & v_{2}^{\left(0\right)} & v_{3}^{\left(0\right)} & v_{4}^{\left(0\right)} & v_{5}^{\left(0\right)} & v_{6}^{\left(0\right)} \end{array}\right]$, and $\bar{\sigma }=\left[\begin{array}{cccccc} \sigma_{11} & \sigma_{12} & \sigma_{13} & \sigma_{22} & \sigma_{23} & \sigma_{33} \end{array}\right]$, where $\sigma_{jk}$ (*j,k* = 1,2,3) is stress component.

Suppose $v^{\left(0\right)}$ stands for a synthetic slip plane with a normal, $n^{\left(0\right)}=\left[\begin{array}{ccc} n_{1}^{\left(0\right)} & n_{2}^{\left(0\right)} & n_{3}^{\left(0\right)} \end{array}\right]$, and a slip line, $s^{\left(0\right)}=\left[\begin{array}{ccc} s_{1}^{\left(0\right)} & s_{2}^{\left(0\right)} & s_{3}^{\left(0\right)} \end{array}\right]$, where $n_{3}^{\left(0\right)}\geq 0$ and $s_{3}^{\left(0\right)}\leq 0$. The permutation of $n^{\left(0\right)}$ and $s^{\left(0\right)}$ in In Eq. (1) does not change the representation of $v^{\left(0\right)}$, which means the existence of a different slip plane, with a normal $s^{\left(0\right)}$ and a slip line $n^{\left(0\right)}$. These slip planes, if they exist, have a similar shear stress sign to the twinned e-plane. Accordingly,

(4a-f) $$\left\{\begin{array}{l} n_{1}^{\left(0\right)}s_{1}^{\left(0\right)}=kv_{1}^{\left(0\right)}\\ n_{1}^{\left(0\right)}s_{2}^{\left(0\right)}+n_{2}^{\left(0\right)}s_{1}^{\left(0\right)}=kv_{2}^{\left(0\right)}\\ n_{1}^{\left(0\right)}s_{3}^{\left(0\right)}+n_{3}^{\left(0\right)}s_{1}^{\left(0\right)}=kv_{3}^{\left(0\right)}\\ n_{2}^{\left(0\right)}s_{2}^{\left(0\right)}=kv_{4}^{\left(0\right)}\\ n_{2}^{\left(0\right)}s_{3}^{\left(0\right)}+n_{3}^{\left(0\right)}s_{2}^{\left(0\right)}=kv_{5}^{\left(0\right)}\\ n_{3}^{\left(0\right)}s_{3}^{\left(0\right)}=kv_{6}^{\left(0\right)} \end{array}\right),$$

where *k* is the scale parameter. The above equation set has a number of seven dependent variables, $n^{\left(0\right)}$ and ${\text{s}}^{\left(0\right)}$ and *k*. Because $n^{\left(i\right)}$ and ${\text{s}}^{\left(i\right)}$ (*i* = 0,1,2) are mutually perpendicular, $v_{1}^{0}+v_{4}^{0}+v_{6}^{0}=0$; therefore, Eqs. (4a), (4d) and (4f) are linearly dependent, too. Eliminating this effect yields a number of five independent equations for the set. Then, both the independent equations and the unit-length equations about $n^{\left(0\right)}$ and ${\text{s}}^{\left(0\right)}$ comprise a well-determined set, from which we solve for all the variables in the following way.

Rewriting Eqs. (4a), (4d) and (4f),

(5a-c) $$\left\{\begin{array}{l} n_{1}^{\left(0\right)}=kv_{1}^{\left(0\right)}/s_{1}^{\left(0\right)}\\ n_{2}^{\left(0\right)}=kv_{4}^{\left(0\right)}/s_{2}^{\left(0\right)}\\ n_{3}^{\left(0\right)}=kv_{6}^{\left(0\right)}/s_{3}^{\left(0\right)} \end{array}\right),$$

After inserting Eqs. (5a) and (5b) into Eq. (4b) and then solving an quadratic equation, we finally have,

(6) s2(0)s1(0)=v±2(0)vv2(0)−2(0)4vv1(0)4(0)2v1(0)=v±2(0)Δ212v1(0),

where the discriminant, $\Delta_{21}$, is expressed as follows,

(7) $$\begin{array}{l} \Delta_{21}={\left[w\left(n_{1}^{\left(1\right)}s_{2}^{\left(1\right)}-n_{2}^{\left(1\right)}s_{1}^{\left(1\right)}\right)-\left(1-w\right)\left(n_{1}^{\left(2\right)}s_{2}^{\left(2\right)}-n_{2}^{\left(2\right)}s_{1}^{\left(2\right)}\right)\right]}^{2}+4w\left(1-w\right).\\ \left(n_{1}^{\left(1\right)}n_{2}^{\left(2\right)}-n_{2}^{\left(1\right)}n_{1}^{\left(2\right)}\right)\left(s_{1}^{\left(1\right)}s_{2}^{\left(2\right)}-s_{2}^{\left(1\right)}s_{1}^{\left(2\right)}\right) \end{array}$$

Whether the discriminant is positive or negative in sign requires additional work. Take as an example the calcite crystal in Fig. 1a, where $n_{2}^{\left(1\right)}=s_{2}^{\left(1\right)}=0$, $n_{3}^{\left(1\right)}=n_{3}^{\left(2\right)}>0$, $s_{3}^{\left(1\right)}=s_{3}^{\left(2\right)}<0$, and $-n_{1}^{\left(1\right)},s_{1}^{\left(1\right)},n_{1}^{\left(2\right)},-n_{2}^{\left(2\right)},-s_{1}^{\left(2\right)},s_{2}^{\left(2\right)}>0$, for simplicity. In this example, $\Delta_{21}={\left[-\left(1-w\right)\left(n_{1}^{\left(2\right)}s_{2}^{\left(2\right)}-n_{2}^{\left(2\right)}s_{1}^{\left(2\right)}\right)\right]}^{2}+4w\left(1-w\right)\left(n_{1}^{\left(1\right)}n_{2}^{\left(2\right)}\right)\left(s_{1}^{\left(1\right)}s_{2}^{\left(2\right)}\right)>0$. That is to say, there are two real solutions to the quadratic equation, because the sign of the discriminant is space-invariant.

Similarly, inserting Eqs. (5a) and (5c) into Eq. (4c) yields,

(8) s3(0)s1(0)=v±3(0)vv3(0)−3(0)4vv1(0)6(0)2v1(0)=v±3(0)Δ312v1(0).

where the discriminant, $\Delta_{31}$, has a positive sign.

According to Eqs. (5), (6) and (8), there are four possible solutions of $s^{\left(0\right)}$ and $n^{\left(0\right)}$:

(9a-d) s(0)=k12v1(0)v+2(0)Δ21v+3(0)Δ31k22v1(0)v+2(0)Δ21v−3(0)Δ31k32v1(0)v−2(0)Δ21v+3(0)Δ31k42v1(0)v−2(0)Δ21v−3(0)Δ31,

and

(10a-d) n(0)=k42v1(0)v−2(0)Δ21v−3(0)Δ31k32v1(0)v−2(0)Δ21v+3(0)Δ31k22v1(0)v+2(0)Δ21v−3(0)Δ31k12v1(0)v+2(0)Δ21v+3(0)Δ31,

where *k_i_* (*i* = 1,2,3,4) is the scale parameter to ensure the unit length of the corresponding vector. The first solution, Eqs. (9a) and (10a), is the permutation of the fourth solution, Eqs. (9d) and (10d); so are the second and third solutions, Eqs. (9b) and (10b) and Eqs. (9c) and (10c). In order to evaluate these possible solutions, we take into account an extreme but simple case of w=1, where $n^{\left(0\right)}=n^{\left(1\right)}$ and $s^{\left(0\right)}=s^{\left(1\right)}$. In this case, by solving $v_{5}^{\left(0\right)}$ in Eqs. (3),(4) for $n^{\left(0\right)}$ and $s^{\left(0\right)}$, we have a seemingly unreal situation of $n^{\left(0\right)}=n^{\left(1\right)}/s^{\left(1\right)}$ and $s^{\left(0\right)}=s^{\left(1\right)}/n^{\left(1\right)}$ for the former pair, and a real situation of $n^{\left(0\right)}=n^{\left(1\right)}$ and $s^{\left(0\right)}=s^{\left(1\right)}$ for the latter pair. That is to say, the latter pair rather than the former pair is accepted as the real solution pair. Furthermore, for the latter pair only the solution with a larger similarity between $n^{\left(0\right)}$ and $n^{\left(1\right)}$ is considered as a unique solution, in the presence of the permutation of $n^{\left(0\right)}$ and $s^{\left(0\right)}$ in $v^{\left(0\right)}$. Such unique solutions for w≠1 are displayed in Fig. 1b and listed in Table 1.

**Table 1:** List of synthetic slip planes in Fig. 1b-c. “0/26.5″ represents dip direction/dip angle for planar data, and bearing/plunge for linear data. Reverse or normal slip sense means the presence of reverse or normal slip component along the slip line.

Pair	Twin and untwin					Weight	Synthetic slip plane		
	Symbol	e-plane	Slip line	Slip sense	Status		Slip plane	Slip line	Slip sense
1	e1	0/26.5	0/26.5	reverse	twin	1.0	0/26.5	0/26.5	reverse
	e2	120/26.5	120/26.5	reverse	untwin	0.9	346.1/26.4	19.1/22.6	reverse
						0.8	340.9/25.9	30.0/17.7	reverse
						0.7	336.9/25.1	40.1/11.9	reverse
						0.6	333.3/23.6	50.1/5.7	reverse
						0.5	330.0/21.0	240.0/0.0	sinistral
2	e1	0/26.5	0/26.5	reverse	twin	1.0	0/26.5	0/26.5	reverse
	e3	240/26.5	240/26.5	reverse	untwin	0.9	13.9/26.4	340.9/22.6	reverse
						0.8	19.1/25.9	330.0/17.7	reverse
						0.7	23.1/25.1	319.9/11.9	reverse
						0.6	26.7/2.6	309.9/5.7	reverse
						0.5	30.0/21.0	120.0/0.0	dextral

In a similar way, for a pair of twinned (*e*_1_) and untwinned (*e*_3_) e-planes in Fig. 1a, we derive the unique solution of $n^{\left(0\right)}$ and $s^{\left(0\right)}$ for a varying weight (Fig. 1c and Table 1).

## Applications

Numerous methods of dynamic analysis have been developed to infer stress from calcite e-twins measured at universal stage. They are based upon two kinds of e-twin data: twinned e-planes [1,4,5,8,12], and both twinned e-planes and untwinned e-planes [6,7,[9], [10], [11]]. They may be categorized as graphic [1] and numerical [4,[6], [7], [8], [9], [10], [11], [12]]. The synthetic slip plane devised in this paper is a hybrid kind of e-twin data, the linear combination of a pair of twinned and untwinned e-planes in a single calcite crystal. It is applicable to graphic and numerical dynamic analysis.

### Graphic dynamic analysis

#### Turner’s [1] C–T method

**Fig. 2:**
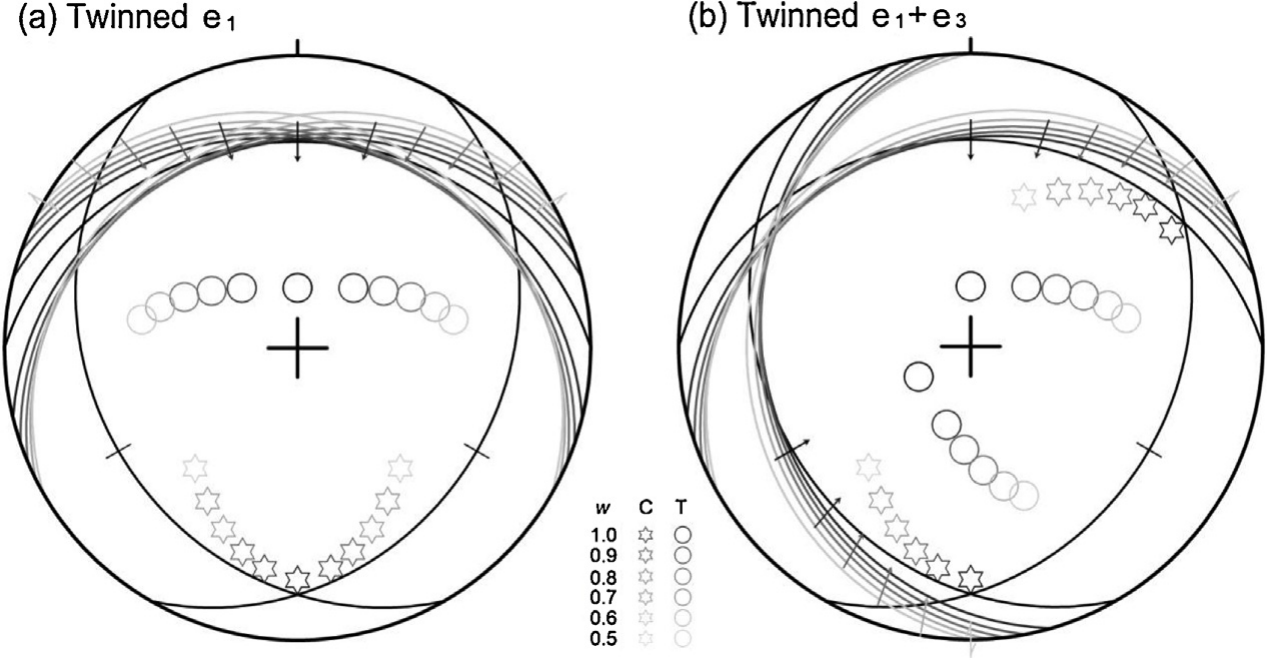
Lower-hemisphere, equal-area projection of the compression (C) and tension (T) axes through applying the C–T method [1] to two examples in a single calcite crystal: (a) one twinned e-plane and two synthetic slip planes, and (b) two twinned e-planes and two synthetic slip planes.

Turner [1] first developed the graphic method that determines the compression (C) and tension (T) axes in an auxiliary plane containing the normal to the twinned e-plane and the gliding line on the plane for each e-twin. This method is directly applicable to the synthetic slip plane. The use of the synthetic plane helps further restrain the extents of the compression and tension axes (Fig. 2).

#### McKenzie’s [14] right dihedral method

**Fig. 3:**
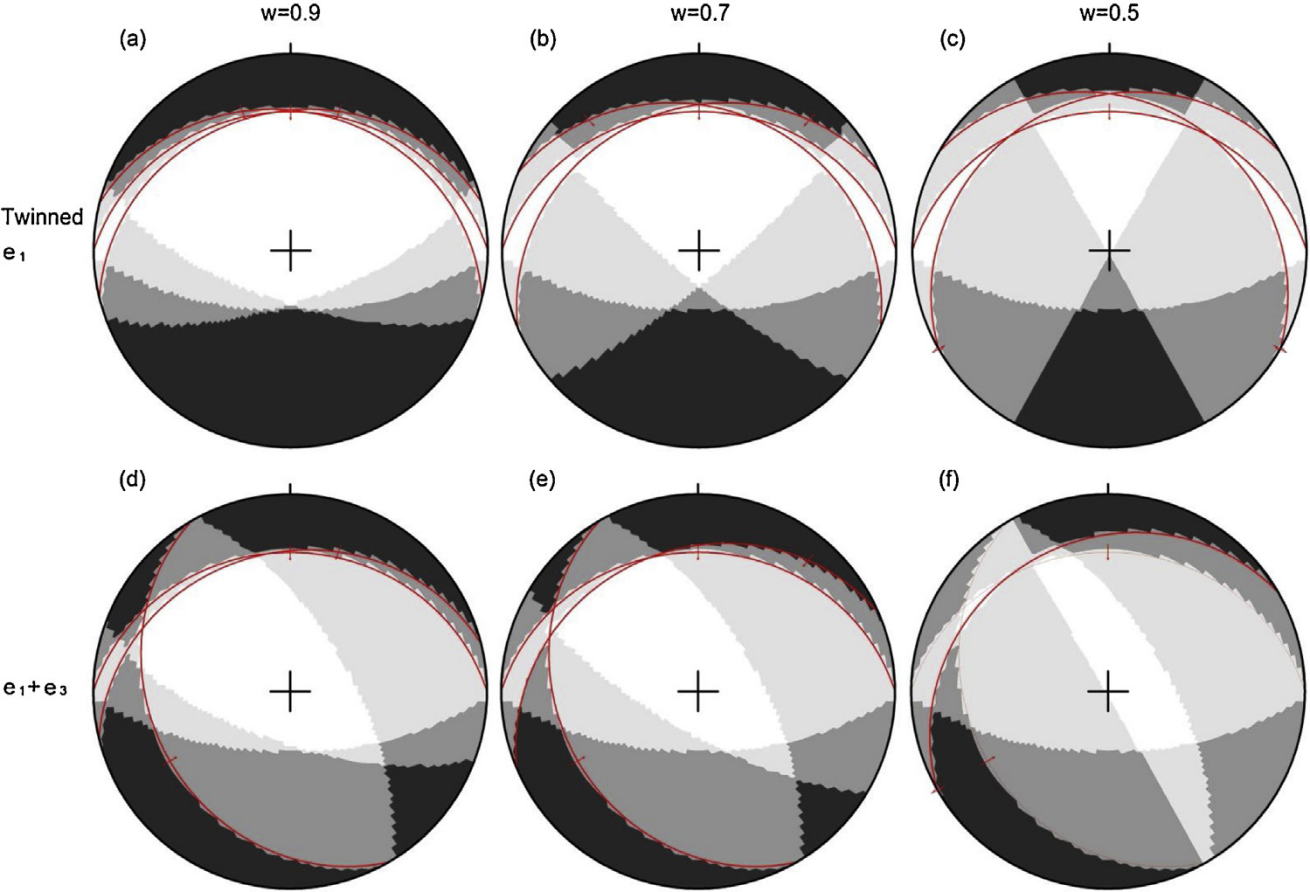
Lower-hemisphere, equal-area projection of the feasible fields for the maximum principal stress axis through applying the right dihedral method to the examples in Fig. 2. (a, d) *w* = 0.9, (b, e) *w* = 0.7 and (c, f) *w* = 0.5 for (a–c) one twinned e-plane (e1) and (d–f) two twinned e-planes (e1+e3). The filled fields have an increasing grey level with the increase in data number. See the text for more explanations.

The right dihedral method for fault data [14] is non-strictly applied to the twinned e-planes (see [5]). The incorporation of the synthetic slip planes into it further reduces the feasible fields of the maximum and minimum principal stress axes. Two examples are taken from a single calcite with an upright *c*-axis (Fig. 1a), for simplicity. The first example has one twinned (*e*_1_) and two untwinned (*e*_2_ and *e*_3_) e-planes (Fig. 2a), and the second example possesses two twinned (*e*_1_ and *e*_3_) and one untwinned (*e*_2_) e-planes (Fig. 2b). The feasible fields have a decreasing extent with the decrease in the weight and the increase in data number (Fig. 3). For w=0.5, the maximum principal stress axis has a relatively narrow range of bearing and a relatively wide range of plunge for the first example (Fig. 3c) and a relatively wide range of bearing and a relatively narrow range of plunge for the second example (Fig. 3f).

### Numerical dynamic analysis

Various methods about numerical dynamic analysis have been developed to obtain either the incomplete deviatoric stress or the reduced stress [4,5] or, more significantly, the complete deviatoric stress [[6], [7], [8], [9], [10], [11], [12]] through solving the optimum problems about the fit or misfit between the calculated and measured twinned and/or untwinned e-planes. The latter methods require the additional knowledge of the value of the critical resolved shear stress to determine the maximum differential stress, as different from the former methods. However, the synthetic slip plane can be incorporated into only the former methods, for example, Spang’s [4] method.

Listed in Table 2 are the results by applying Spang’s [4] method to examples shown in Fig. 2. For the first example, the maximum and minimum principal stresses are within the feasible fields obtained using the right dihedral method (Fig. 3a–c), regardless of the weight. The intermediate principal stresses have a constant orientation. With the decrease in the weight, the maximum or minimum principal stress has an increasing or decreasing plunge, and the stress ratio decreases.

**Table 2:** Stress results by applying Spang’s [4] to the examples shown in Fig. 2. SSP is the abbreviation of synthetic slip plane. The stress ratio is the ratio of $\left(\sigma_{2}-\sigma_{3}\right)$ to $\left(\sigma_{1}-\sigma_{3}\right)$, where $\sigma_{1}$, $\sigma_{2}$ and $\sigma_{3}$ are the maximum, intermediate and minimum principal stress magnitudes.

Data	Weight	Principal stress orientations			Stress ratio
		Maximum	Intermediate	Minimum	
1 e-twin		180.0/18.5	90.0/0.0	0.0 /71.5	0.50
1 e-twin + SSPs	0.9	180.0/19.6	90.0/0.0	0.0/70.4	0.47
	0.7	180.0 /22.8	90.0 /0.0	0.0/67.2	0.38
	0.5	180.0/27.1	90.0/0.0	0.0/62.9	0.25
2 e-twins		30.0/0.0	120.0/15.5	300.0/74.5	0.79
2 e-twins + SSPs	0.9	30.0/0.0	120.0/10.3	300.0/79.7	0.70
	0.7	30.0/0.0	120.0/3.4	300.0/86.6	0.56
	0.5	30.0/0.0	300.00/12.9	120.0/77.1	0.44

For the second example, the maximum principal stress is always within the feasible fields (Fig. 3d–f); so is the minimum principal stress only for a larger value of weight. The maximum principal stresses have a constant orientation. With the decrease in the weight, the intermediate or minimum principal stress has a decreasing or increasing plunge, and the stress ratio decreases.

## Concluding remarks

In this paper the synthetic slip plane is defined as the linear combination of a pair of twinned and untwinned e-planes in a single calcite crystal. It is independent of either twinned e-plane or untwinned e-plane. This auxiliary slip plane can be used together with the twinned e-planes to better determine stress in both graphic dynamic analysis and numerical dynamic analysis. However, in the latter analysis, this new kind of data is at present incorporated into only the existing methods that solve for the reduced stress.
